# Supplementary material for: Collaborative intelligence in AI: Evaluating the performance of a council of AIs on the USMLE
Source: PLOS Digit Health. 2025 Oct 9;4(10):e0000787. doi: 10.1371/journal.pdig.0000787 (PMC12510544; doi:10.1371/journal.pdig.0000787)
Supplement: S2 File — (ZIP) [file pdig.0000787.s002.zip › S2 Data Raw data files containing unprocessed question inputs and LLM outputs including transcripts of deliberations/STEP3_Question-120-1_Type-MC-NJ.docx]

**Facilitator** **[Round 1]:** A 25-year-old man comes to the emergency department because he developed chest pain and shortness of breath 1 hour ago, shortly after snorting cocaine for the first time. He rates the chest pain as a 7 on a 10-point scale and notes that the pain is radiating down his left arm. Medical history is unremarkable and the patient takes no medications or any other illicit drugs. He is 178 cm (5 ft 10 in) tall and weighs 70 kg (154 lb); BMI is 22 kg/m². The patient is diaphoretic. Vital signs are temperature 37.5°C (99.5°F), pulse 110/min, respirations 16/min, and blood pressure 200/100 mm Hg. Pulse oximetry on room air shows an oxygen saturation of 95%. Pupils are equal, round, and reactive to light and accommodation. Lungs are clear to auscultation and percussion. Auscultation of the heart discloses an audible S1 and S2. There is no edema, cyanosis, or clubbing of the digits. The patient is fully oriented. He is treated with supplemental oxygen, a 325-mg aspirin tablet, and intravenous nitroglycerin and lorazepam. Despite therapy, he continues to have chest pain and shortness of breath. ECG shows sinus tachycardia with no ST-segment or T-wave abnormalities. Which of the following is the most appropriate additional pharmacotherapy to initiate at this time? (A) Carvedilol (B) Furosemide (C) Metoprolol (D) Phentolamine (E) Streptokinase

**AI_D:** 1) RESTATING THE QUESTION: A 25-year-old man is experiencing chest pain and shortness of breath after snorting cocaine for the first time. Initial treatment with oxygen, aspirin, nitroglycerin, and lorazepam has not alleviated his symptoms, and his ECG shows sinus tachycardia without ST-segment or T-wave abnormalities. We need to determine what additional pharmacotherapy would be most suitable for him. 2) PLANNING: We know that cocaine is a potent stimulant that causes vasoconstriction and increases heart rate and blood pressure. The symptoms that the patient is experiencing (chest pain radiating down the left arm and shortness of breath) could be suggestive of myocardial ischemia (inadequate blood flow to the heart muscle). Given that, we could make the assumption that additional pharmacotherapy should target cocaine's effects on the cardiovascular system. 3) CLINICAL REASONING: There are several medications listed as options for additional treatment, but their indicated uses vary widely. For instance, Carvedilol and Metoprolol are beta-blockers primarily used to decrease heart rate and blood pressure; however, using beta-blockers in the context of cocaine use, can paradoxically increase vasoconstriction. Alternatively, Furosemide is a diuretic used to reduce fluid volume where fluid overload is not a current issue in our patient. Streptokinase, a thrombolytic agent may not be suitable given the fact our patient's ECG doesn't show a clear sign of ST-segment elevation myocardial infarction (STEMI), which thrombolytic therapy would be indicated for. Meanwhile, Phentolamine, a non-selective alpha blocker, can be used to counteract the vasoconstrictive effects of cocaine, therefore improving blood flow to the heart and other vital organs without the risk of unopposed alpha stimulation. 4) CONCLUDING: From this clinical reasoning, we can conclude that Phentolamine aligns most closely with our initial assumptions in terms of counteracting the pathophysiological impacts of cocaine use on the cardiovascular system. In other words, it offers a potential solution to alleviate the myocardial ischemia by counteracting the vasoconstrictive effects of cocaine. 5) RESPONSE: Therefore, the most appropriate additional pharmacotherapy to initiate at this time would be Phentolamine (choice D).

**AI_C:** RESTATING THE QUESTION: A 25-year-old man, with no notable medical history and no regular medications, has developed chest pain radiating down his left arm and breathlessness after snorting cocaine. Despite attempts to decrease the chest pain and breathing difficulties with interventions like supplemental oxygen, aspirin, nitroglycerin, and lorazepam, his symptoms continue. His blood pressure is significantly high, and his heart rate is above normal (sinus tachycardia). Notably, his ECG shows no ST-segment or T-wave abnormalities, hinting that the symptomatology may not be due to a typical myocardial infarction. The question is asking which medication should be given next to this patient. PLANNING: We first assume that the patient's symptoms are secondary to his cocaine use. Cocaine causes an influx of catecholamines, leading to intense vasoconstriction and increased myocardial oxygen demand - this could explain the onset of the patient's symptoms. Hydrostatic pressure within the vessels likely elevated secondary to intense vasoconstriction may give rise to symptoms such as dyspnea and chest pain even in the absence of pulmonary edema or overt signs of heart failure. Cocaine-induced hypertension should be treated differently than typical cases of hypertension: Direct vasodilators and Beta-blockers can cause unopposed alpha-adrenergic stimulation. CLINICAL REASONING: Therefore drugs that antagonize alpha-adrenergic receptors would be clinically prioritized to pursue. It means we should look into medications that can help decrease the sympathetic activity stimulated by cocaine on a vascular level, especially an alpha-adrenergic antagonist. This will help counteract the vasoconstrictive elements of the increased adrenergic (norepinephrine) activity seen with cocaine use. By reversing the vasoconstriction, we could potentially alleviate the patient's symptoms. CONCLUDING: Given the information supplied, amongst the available options, Phentolamine, an alpha-adrenergic antagonist, seems to best align with the clinical thought process outlined. RESPONSE: Indeed, Phentolamine (D) is the most appropriate additional pharmacotherapy to initiate at this time for treatment of hypertension and cardiovascular symptoms induced by cocaine. It will act to negate the heightened sympathetic activity caused by cocaine misuse, and thereby alleviate the vasoconstriction secondary to the alpha-adrenergic stimulation.

**AI_B:** (1) RESTATING THE QUESTION: The query seeks to establish the most suitable pharmacological treatment to administer to a 25-year old male patient who, after using cocaine for the first time, developed chest pain and acute shortness of breath, symptoms characteristic of cardiovascular conditions. Despite initial treatments, his symptoms persist, demanding further pharmacological intervention. Cocaine is a potent central nervous system stimulant that causes an extraordinary release of norepinephrine, dopamine, and serotonin in the brain leading to euphoria, increased alertness, and increased systolic and diastolic blood pressure due to its vasoconstrictive properties. (2) PLANNING: Cocaine-induced chest pain could be a result of coronary vasospasm, acute myocardial ischemia, or even myocardial infarction. However, in this case, the ECG showed sinus tachycardia without ST-segment or T-wave abnormalities, which makes myocardial infarction less likely. This narrows assumption to coronary vasospasm or acute myocardial ischemia induced by cocaine use. Even though some beta-blockers may seem appropriate for chest pain under normal circumstances, their use could potentially cause "unopposed alpha stimulation" in a patient who has recently used cocaine. The choice lies mostly between alpha-blockers and direct-acting vasodilators in this scenario. (3) CLINICAL REASONING: Cocaine’s effect mainly results from its ability to block the reuptake of norepinephrine in the synaptic cleft, resulting in sustained activation of alpha-1 adrenergic receptors, causing vasoconstriction, increased systemic vascular resistance, and hypertension. Beta-blockers (like Carvedilol and Metoprolol), in this case, could increase the alpha-1 stimulation (unopposed alpha stimulation), leading to more intense vasoconstriction and hypertension. Furosemide, a diuretic, is not used as a first line-drug in acute coronary syndrome management, and Streptokinase, a thrombolytic agent, is used in confirmed myocardial infarction, which is unlikely in this scenario as commented before. (4) CONCLUDING: Based on the clinical context, using the process of elimination, reinforcing the pathophysiology of cocaine use, and urgency of managing acute cardiac complications, it is therefore crucial to relax the vasculature to alleviate these severe symptoms. (5) RESPONSE: Therefore, the most appropriate next step in pharmacotherapy is use of (D) Phentolamine, an alpha-adrenergic antagonist, which could counteract the norepinephrine-induced vasoconstriction caused by cocaine intake. This would facilitate relievement of chest pain and shortness of breath by reversing the vasoconstrictive effects of cocaine on coronary arteries.

**AI_E:** (1) RESTATING THE QUESTION: A young man, who has just used cocaine for the first time, is experiencing severe chest pain and difficulty breathing. Despite receiving initial treatments of supplemental oxygen, aspirin, nitroglycerin, and lorazepam, his pain and breathing problems persist. His vital signs indicate a heightened heart rate, and his blood pressure is significantly elevated. His ECG, however, does not show any abnormalities in ST-segments or T-waves. Cocaine is a potent stimulant drug that can significantly affect cardiovascular function and is associated with severe life-threatening complications such as myocardial infarction, arrhythmias, hypertension, and aortic dissection. The question is asking for the best pharmacotherapy to give in this situation. (2) PLANNING: These symptoms combined with his recent cocaine use indicate a potential acute cocaine toxicity leading to potential cardiovascular complications. Carvedilol, furosemide, metoprolol, phentolamine, and streptokinase all have their roles in cardiovascular management. Assumptions here include the concept that the symptoms are purely due to the cocaine and not an underlying condition, that the cocaine was not laced with other substances, and that the initial treatments have had no effect. The best pharmacotherapy would effectively manage the high blood pressure and heart rate caused by cocaine to prevent further harm. (3) CLINICAL REASONING: Since clinical interventions have failed to alleviate this patient's chest pain and shortness of breath and the ECG does not indicate a myocardial infarction (no ST-segment or T-wave abnormalities), it is plausible that this patient may have cocaine-induced severe hypertension and vasoconstriction, rather than acute coronary syndrome. Cocaine can lead to significant vasoconstriction due to increased levels of circulating catecholamines. Beta blockers like carvedilol and metoprolol could potentially exacerbate cocaine-induced coronary spasm and hypertension due to unopposed alpha-adrenergic receptor stimulation, so they should be avoided. Furosemide is a diuretic, typically used to treat fluid overload conditions like heart failure, and not first-line for managing acute hypertension. Streptokinase is a thrombolytic and would be considered if the patient exhibited ECG changes suggestive of ST-elevation myocardial infarction (STEMI), which is not present in this context. (4) CONCLUDING: Phentolamine, an alpha-blocker, is a practical choice in this instance. It interferes with the vasoconstrictive effects of cocaine by blocking alpha-adrenergic receptors. Therefore, one can expect phentolamine to alleviate the severe hypertension and chest pain induced by cocaine. (5) RESPONSE: Thus, the most appropriate additional pharmacotherapy to initiate at this time would be (D) Phentolamine, due to its role in blocking the alpha-adrenergic receptors, which counteracts the vasoconstrictive effects of the cocaine, offering potential alleviation of the hypertension and chest pain.

**AI_A:** Before I delve into deciphering the best pharmacotherapy in this case, it is crucial to understand the key elements and aspects of this clinical presentation. A young man, following his first-ever use of cocaine, has come to the emergency department with chest pain and shortness of breath. This pain is severe in nature, rated 7/10, and is radiating down his left arm. He doesn't have a remarkable medical history and is not on any regular medications or other illicit drugs. His physical examination reveals tachycardia, elevated blood pressure, and diaphoresis - all potential signs of a cardiac event. However, his ECG doesn't show any definitive evidence of cardiac ischemia. Despite being treated with supplemental oxygen, aspirin, IV nitroglycerin, and lorazepam, his symptoms continue. The main question here is to identify the most appropriate additional pharmacotherapy. To approach this, we need to understand the potential pathophysiological processes that may be instigated by cocaine use, and how each of the treatment options proposed will address those processes. Cocaine is known to increase sympathetic activity, causing surges in heart rate, contractility, and blood pressure, through its inhibition of catecholamine reuptake in the synaptic cleft. Cocaine can also cause intense vasoconstriction via its inhibitory effect on nitric oxide synthetase, leading to reduced NO production in the vascular endothelium. This, coupled with the heightened adrenergic activity causing elevated blood pressure, can precipitate cardiac ischemia, despite no obstructive coronary artery disease being present. This syndrome is known as cocaine-induced vasospasm or cocaine-induced myocardial ischemia. The fact that this patient has chest pain occurring at rest, in the setting of one-hour cocaine use, with an absence of ST-T wave changes, strongly supports this diagnosis. Understanding this condition leads us to make assumptions regarding what kind of medication would work best. We need a drug that can counteract the intense vasoconstriction caused by cocaine. Looking at the treatment options, we have Carvedilol, Furosemide, Metoprolol, Phentolamine, and Streptokinase. It is important to know that beta-blockers (Carvedilol and Metoprolol) would not be ideal in this setting. They could cause an unopposed alpha-adrenergic stimulatory effect exacerbating the cocaine-induced vasoconstriction. Furosemide, a diuretic, isn't indicated here as the patient doesn't show signs of fluid overload. Streptokinase, a fibrinolytic drug, is typically employed in confirmed ST-elevation myocardial infarction (STEMI), which is not plausible here due to the lack of ECG changes. On the other hand, Phentolamine is an alpha-adrenergic antagonist. It blocks alpha receptors, thus inhibiting the vasoconstriction caused by stimulant-induced sympathetic overactivity. This function makes Phentolamine a potential candidate. Using our clinical reasoning, it seems that Phentolamine is the most clinically appropriate choice. It targets the crux of the problem - cocaine-induced vasoconstriction - by antagonizing the alpha-adrenergic effects. To sum up, in a young patient with severe chest pain after using cocaine for the first time, with no ECG changes indicative of ischemia or infarction, and where other medications have not alleviated the pain, the drug of choice that directly counters the cocaine-induced pathophysiology of coronary vasospasm, is Phentolamine, an alpha-adrenergic antagonist. Therefore, the most appropriate additional pharmacotherapy to initiate in this patient, at this time, is (D) Phentolamine.

**Facilitator** The consensus statement is as follows: A 25-year-old man, after his first-ever use of cocaine, has presented with severe chest pain and shortness of breath. Despite initial treatment with supplemental oxygen, aspirin, IV nitroglycerin, and lorazepam, his symptoms persist. His physical examination reveals tachycardia, elevated blood pressure, and diaphoresis, but his ECG doesn't show any definitive evidence of cardiac ischemia. Cocaine is known to increase sympathetic activity, causing surges in heart rate, contractility, and blood pressure, and can cause intense vasoconstriction. This, coupled with the heightened adrenergic activity causing elevated blood pressure, can precipitate cardiac ischemia, despite no obstructive coronary artery disease being present. This syndrome is known as cocaine-induced vasospasm or cocaine-induced myocardial ischemia. Given the treatment options of Carvedilol, Furosemide, Metoprolol, Phentolamine, and Streptokinase, beta-blockers (Carvedilol and Metoprolol) would not be ideal as they could cause an unopposed alpha-adrenergic stimulatory effect exacerbating the cocaine-induced vasoconstriction. Furosemide, a diuretic, isn't indicated here as the patient doesn't show signs of fluid overload. Streptokinase, a fibrinolytic drug, is typically employed in confirmed ST-elevation myocardial infarction (STEMI), which is not plausible here due to the lack of ECG changes. Phentolamine, an alpha-adrenergic antagonist, blocks alpha receptors, thus inhibiting the vasoconstriction caused by stimulant-induced sympathetic overactivity. This function makes Phentolamine a potential candidate. Therefore, the most appropriate additional pharmacotherapy to initiate in this patient, at this time, is Phentolamine. It targets the crux of the problem - cocaine-induced vasoconstriction - by antagonizing the alpha-adrenergic effects.
